# Intratracheal Medication

**Published:** 1902-10

**Authors:** Edgar A. Forsyth

**Affiliations:** Buffalo, N. Y., Laryngologist, Rhinologist and Otologist to the Emergency Hospital, 326 Franklin Street.


### Intratracheal Medication.

By EDGAR A. FORSYTH, M. D., Buffalo, N. Y.,
Laryngologist, Rhinologist and Otologist to the Emergency Hospital.

DISEASES of the larynx and of the respiratory organs have been treated by intratracheal medication since 1838 when Horace Green, of New York, passed a small sponge saturated with a solution of nitrate of silver through the glottis into the trachea. In 1840 he reported 15 cases of severe laryngitis and bronchial diseases which had been cured by this means. In 1854 he injected into the lungs through an elastic tube passed through the larynx into the bronchi 1½ to 2 drams of strong nitrate of silver solution; his patients grew stronger, breathed better and increased in weight. From October, 1854, to February, 1856, he treated 106 cases, of which 71 were cases of tuberculosis. Of the tubercular cases, 32 were considered as cases of advanced tuberculosis, and 39 as cases of early tuberculosis. Of the advanced cases 25 were more or less improved, their lives being prolonged; 7 were not improved at all. Of the 39 cases of incipient tuberculosis, 12 had apparently recovered at the time the report was written and 5 more were nearly well; of the remaining 22 cases, 17 were greatly improved, 3 were moderately benefited and 3 were not improved at all. Of the 28 cases of bronchitis 16 were cured and all of the others greatly benefited. In 6 cases of asthma treated by injections the disease was entirely removed in all but one.

Several years later Horace Green published another paper in which he said he was using milder solutions and added "such has been the amount of success which has continued to attend this plan of treatment that I am now ready to affirm, after an experience of many years in a field of observation unusually large, that if I were required to relinquish all other known therapeutic measures or topical medication, I should choose the latter with hygienic means alone, in preference to the entire class of remedies ordinarily employed in the treatment of these diseases. During the three or four years since my report of 106 cases, I have treated large numbers of patients afflicted with chronic laryngeal and bronchial diseases, with asthma and tuberculosis, and the success which continues to attend this practice has served to increase greatly my confidence in this measure as a therapeutic agent."

The advantage of the intratracheal injections over the usual methods of treatment in tuberculosis lies in the fact that the antiseptics used enter at once the field of their labors unaltered and penetrate to the farthest limit of the respiratory space and coming in immediate contact with the toxins as they are generated bring about the beneficial results without interfering with the digestive organs.

Colin Campbell, in the *Lancet* of September 11, 1897, says there can be no doubt about the absorption of the drugs by the lung tissue, as he has injected into the trachea of patients each week one quart of fluid. If not absorbed, what becomes of it? Experiments made by Dr. Pernice show that liquids injected into the trachea run down the posterior surface and are thence evenly distributed through all the bronchial twigs and are absorbed by the pulmonary veins or by the lymphatic vessels and thus exert both a local and a systemic action.

Dr. C. G. Coakley in the *Medical Record* of February 8, 1896, reports results of a series of experiments conducted at the Loomis laboratory. He made a solution of India ink, filtered it and injected ten cubic centimeters of the filtered solution into the trachea of rabbits and the animals were killed in from fifteen minutes to two hours. It was found in every case that not a trace of the pigment could be found in the trachea, bronchi or alveoli, but that all had been absorbed.

I began giving intratracheal injections in February, 1897, and since that time have given a great many and have never seen any bad effects. Spasms of the glottis have never occurred and the patients are not opposed to taking them, as a rule. I remember only one patient who said he could not take them.

Most of them experience so much relief they say they wish they could have them more often. With this treatment the expectoration becomes greatly reduced in quantity and much less offensive; the purulent element disappears and expectoration resembles the frothy expectoration of simple bronchitis; fever and night sweats disappear and patient gains in weight.

Quaiacol and similar preparations frequently produce ill-effects when taken by the mouth and have to be discontinued; opiates are not required, as intratracheal injections accomplish all that can be done by their use. J. A. Thompson, in the *Therapeutic Gazette* of October 15, 1898, says that in the hospital for consumptives, with which he is connected, cough is never treated by opiates if the patient is able to go to the treatment room and be given a tracheal injection. These injections he finds relieve the cough more satisfactorily than any internal medication. In cases in which the cough is so excessive as to cause vomiting and nutrition is in consequence rapidly failing the placing in trachea of 1 to 3 drams of a 2 per cent. solution of menthol, an hour before eating, will permit of both the ingestion and assimilation of the essential nutriment. By this method the detrimental effects of the use of opium and its derivatives to control the cough are entirely avoided.

The diseases most suitable to this form of treatment are pulmonary and laryngeal tuberculosis, pulmonary and laryngeal syphilis, chronic bronchitis, inflammation of trachea, asthma and bronchiectasis. Lactic acid is used mostly for laryngeal tuberculosis when there is ulceration, but these applications are painful and pain often lasts for hours. Patients dread the treatment, but the intratracheal injections give such relief at once that the patient enjoys taking them.

R. Lake advises intratracheal injections for granular cords and superficial excoriations or ulceration, and reported to the Laryngological Society of London, June 8, 1898, a case of cured laryngeal tuberculosis. There was an ulcer on each cord and one on anterior part of cricoid cartilage. He gave daily intratracheal injections of a solution of naphthalene, 3 per cent.; oil of cinnamon, ½ per cent., in parolene.

W. Freudenthal has been using intratracheal injections of menthol-orthoform emulsion with good success in regard to pain; the ulcers will heal but return to disappear again after treatment.

The solutions I use are menthol 7½ to 15 per cent., guaiacol,

$\frac{1}{2}$ to 3 per cent. in benzoinol or alboline. Europhen, 1 to 2 per cent., may be used in place of the guaiacol. The patient will be nervous about first few injections, so I spray or apply with applicator a 2 per cent. solution of cocain to the larynx. After a few injections this may be stopped. I use instrument suggested by Joseph Muir which has a capacity of half an ounce. One to 4 drams may be used. Muir in one case of purulent bronchitis administered an ounce at one sitting.

Injections are made about the same as laryngeal applications. The patient grasps the tongue with the right hand, drawing it forward, at the same time throwing the head back and opening the mouth as widely as possible. The operator holds the laryngeal mirror in one hand and syringe in the other and proceeds as though he were about to make a laryngeal application. As soon as the tube enters the cavity of the larynx the epiglottis is pulled slightly forward, the patient is instructed to breathe, the cords separate, the tube enters between the cords, and the syringe is emptied of the desired amount. By pointing the instrument to either side the bulk of the medication may be made to enter either the right or the left bronchus. It is not necessary to introduce the end of the syringe below the vocal cords, as results are just as good when end of syringe is placed above them and then medication reaches all parts of larynx as well as trachea and lungs.

J. L. Barton says all the cases of tuberculosis he treated by this method were markedly benefited, while those having laryngotracheitis and bronchitis were rapidly restored to health, and one case of asthma was greatly improved.

My results with intratracheal injections have been very encouraging and certainly will cause me to use it in preference to any other in cases of laryngeal and pulmonary tuberculosis. My patients experience great relief from the injections and increase in weight; some of them have gained as much as seventeen pounds, while only one lost in weight, and he had been losing rapidly for some time. While under my care he lost three pounds, but he was working out of doors at night in all kinds of weather. He did not remain under my care more than two months, as he lost so much sleep by coming to my office he had to give up treatments. It was a case that was very unsatisfactory, as his visits were not frequent enough to do him any good.

I do not believe intratracheal injections will cure every case of laryngeal or pulmonary tuberculosis, but do believe it is the best method of treating these diseases we possess at the present time. I know I have had ulcers heal and the patient improve in every way, but these cases are sometimes very difficult to manage, as they will not come to your office when they are feeling well and are able to go to work, as one case did after treatment. Before I began injections he had been unable to work for one year, and had been sick for eighteen months; complained of cough, pain in the chest, shortness of breath, pain in the throat, hoarseness; swallowing either solids or liquids was painful, slight elevation of temperature; pulse, 104; lost a great deal in weight. Examination showed pulmonary tuberculosis with ulcers in the larynx. His wife died twelve years ago with laryngeal tuberculosis, and one brother died of tuberculosis. He received his injections for six months, when he said he had increased in strength, coughed less and felt so well he wanted to go to work. He was a carpenter and went to work on a new house at a summer resort on the lake shore. He began work in the spring and slept in the house, which was only partly finished, during very bad weather and contracted a cold, which made him come back to me for further treatment, but as soon as he felt better he discontinued treatment and went to work again. I saw him once after that and he was feeling quite well.

The most interesting case I have treated by intratracheal injections is that of a man 33 years old, a tailor. When he came to me he gave the following history:

Had been hoarse for over one year, more marked during the last six months; had been taking injections of tuberculin. He began treatment with me March 23, 1900. Examination of his lungs showed pulmonary tuberculosis; larynx showed infiltration and ulcers; sputum examined showed tubercle bacilli. I treated him until April 9, 1900, by local applications without much relief, at which time he discontinued his visits; he resumed treatment May 10, 1900, when he said his condition was much worse than when I saw him last. He had a great deal of pain in his chest, coughs a great deal, spits blood, raises a great deal of purulent sputum. I began giving him intratracheal injections of menthol, europhen and alboline. I gave the injection every other day, using 2 drams, and on alternate days used 50 per cent. lactic acid. The tuberculin was stopped. On May 31 he had gained 4½ pounds, coughs less, spits no blood and feels much stronger. He was very faithful about taking his treatment and as he received more benefit from the injections than from the lactic acid I stopped it after a short time and gave him the injections every day. He gained in every way; ulcers in larynx healed up, no cough or pain in the chest; he gained in all eleven pounds and so much in strength that he was able to do a great deal of work; in fact, as much as ever—lift and carry large, heavy bundles.

I kept him under my care after he noticed all these improvements and gave him two or three injections a week, as he was afraid he might not be able to get along without them. After a while he discontinued treatment, and I did not see anything of him until November 19, 1901, when he came to my office to report that he was still feeling well. I had his sputum examined and report showed no bacilli. I saw him last, August 31, 1902, when he was in good health and working hard every day.

I believe this case demonstrates what intratracheal injections will do for laryngeal and pulmonary tuberculosis. He also received tonics and was advised to be out in the air as much as possible.

I know more cases of tuberculosis would recover if they were given intratracheal injections, their nutrition carefully looked after, and the rules of hygiene closely followed.

326 Franklin Street.

